# 

**Published:** 1906-08

**Authors:** 


### Contributors to Vol. LXII.

AUGUST, 1906—JULY, 1907.

---

Beahan, Albert L. . . . . . . . . . . . . . . . . . . . . . . . . . . . . Canandaigua
Birchmore, Woodbridge Hall . . . . . . . . . . . . . . . . . . . . . Brooklyn
Boswell, Charles O. . . . . . . . . . . . . . . . . . . . . . . . . . . . Rochester
Brown, C. W. M. . . . . . . . . . . . . . . . . . . . . . . . . . . . . . Elmira
Brown, William M. . . . . . . . . . . . . . . . . . . . . . . . . . . . Rochester
Brush, Edward N. . . . . . . . . . . . . . . . . . . . . . . . . . . . . Baltimore
Burnham, M. P. . . . . . . . . . . . . . . . . . . . . . . . . . . . . . Raybrook
Cannaday, John Egarton . . . . . . . . . . . . . . . . . . Hansford, W. Va
Clinton, Marshall . . . . . . . . . . . . . . . . . . . . . . . . . . . . . Buffalo
Cook, Robert G. . . . . . . . . . . . . . . . . . . . . . . . . . . . . . Rochester
Dixon, Samuel C. . . . . . . . . . . . . . . . . . . . . . . . . . . . . Harrisburg
Frazier, Charles H. . . . . . . . . . . . . . . . . . . . . . . . . . . Philadelphia
Fronczak, Francis E. . . . . . . . . . . . . . . . . . . . . . . . . . . . Buffalo
Frye, Maud J. . . . . . . . . . . . . . . . . . . . . . . . . . . . . . . . . Buffalo
Gardner, James A. . . . . . . . . . . . . . . . . . . . . . . . . . . . . Buffalo
Greene, Dewitt C. . . . . . . . . . . . . . . . . . . . . . . . . . . . . . Buffalo
Gregg, Milton E. . . . . . . . . . . . . . . . . . . . . . . . . . . . Elbridge, N. Y
Goldberg, Sigmund . . . . . . . . . . . . . . . . . . . . . . . . . . . . Buffalo
Gould, George M. . . . . . . . . . . . . . . . . . . . . . . . . . . . Philadelphia
Hayd, Herman E. . . . . . . . . . . . . . . . . . . . . . . . . . . . . . Buffalo
Hubbell, Alvin A. . . . . . . . . . . . . . . . . . . . . . . . . . . . . . Buffalo
Jacobson, Nathan . . . . . . . . . . . . . . . . . . . . . . . . . . . . . Syracuse
Jameson, Thomas . . . . . . . . . . . . . . . . . . . . . . . . . . . . . Rochester
Jenkins, J. F. T. . . . . . . . . . . . . . . . . . . . . . . . . . . . . Los Angeles
Johnson, William D. . . . . . . . . . . . . . . . . . . . . . . . . . . . Batavia
King, James E. . . . . . . . . . . . . . . . . . . . . . . . . . . . . . . . Buffalo
Krauss, William C. . . . . . . . . . . . . . . . . . . . . . . . . . . . . Buffalo
Langdon, H. H. . . . . . . . . . . . . . . . . . . . . . . . . . . . . . New York
LeBreton, Prescott . . . . . . . . . . . . . . . . . . . . . . . . . . . . . Buffalo

Levy, I. H. .......... Syracuse
Lewis, Denslow .......... Chicago
Lewis, F. Park .......... Buffalo
Low, Frank W. .......... Buffalo
Lucid, M. M. .......... Cortland
McKee, E. S. .......... Cincinnati
Macpherson, J. D. .......... Akron
Marchand, Charles .......... New York
Morse, John Lovett .......... Boston
Norton, J. Pease .......... New Haven
Park, Roswell .......... Buffalo
Pilcher, James Evelyn .......... Carlisle
Putnam, James Wright .......... Buffalo
Rooker, A. M. .......... Buffalo
Rose, L. W. .......... Rochester
Smith, Eugene A. .......... Buffalo
Smith, Herbert E. .......... New Haven
Stover, Charles .......... Amsterdam
Thoma, Fridolin .......... Buffalo
Totman, David M. .......... Syracuse
Vander Veer, Albert .......... Albany
Wallace, W. L. .......... Syracuse
Warbrick, James C. .......... Chicago
Wilder, Burt G. .......... Ithaca
Williams, Henry T. .......... Rochester
Wilson, Nelson W. .......... Buffalo
Wright, Adam H. .......... Toronto
Young, J. C. .......... Cuba, N. Y

Medical Association of Central New York
Medical Society of the County of Erie

